# Supplementary material for: A Likelihood Approach for Real-Time Calibration of Stochastic Compartmental Epidemic Models
Source: PLoS Comput Biol. 2017 Jan 17;13(1):e1005257. doi: 10.1371/journal.pcbi.1005257 (PMC5240920; doi:10.1371/journal.pcbi.1005257)
Supplement: S1 File — (TAR.GZ) [file pcbi.1005257.s014.tar.gz › HSPH_Online-SI-Revision/PF08/n10-severe/n10-severe_PF_summary.pdf]

peak -8	R0	$1/\theta_2$	inf	inflog	newly	newlylog	Reff	AttackRate
mean	21.6	156.9	1274.5	0.8	350.	0.4	19.7	26.9
median	18.1	141.8	515.6	0.7	112.8	0.3	16.3	29.2
Quantile .1	4.9	83.7	170.4	0.4	36.8	0.1	6.1	11.1
Quantile .9	39.3	255.4	2490.7	1.3	619.7	0.8	35.4	40.6
46	0	32						
length	50							

peak -4	R0	$1/\theta_2$	inf	inflog	newly	newlylog	Reff	AttackRate
mean	17.	173.2	111.9	0.3	33.	0.2	14.4	27.
median	16.3	147.5	95.9	0.3	33.4	0.2	12.5	26.5
Quantile .1	4.2	84.7	39.	0.1	19.7	0.1	5.3	10.3
Quantile .9	31.3	262.6	192.8	0.4	44.9	0.3	26.4	44.2
35	0	17						
length	50							

peak	R0	$1/\theta_2$	inf	inflog	newly	newlylog	Reff	AttackRate
mean	35.6	187.9	171.7	0.4	15.4	0.1	14.	50.5
median	33.3	164.8	157.6	0.4	12.8	0.1	12.1	49.8
Quantile .1	7.4	87.3	62.2	0.2	6.8	0.	5.	29.8
Quantile .9	70.4	314.6	302.1	0.6	26.	0.1	25.	68.7
2	0	0						
length	50							

peak +4	R0	$1/\theta_2$	inf	inflog	newly	newlylog	Reff	AttackRate
mean	30.2	169.	454.	0.6	101.9	0.3	10.	51.7
median	23.1	133.4	276.6	0.6	69.5	0.2	7.7	49.5
Quantile .1	9.2	85.4	132.2	0.4	20.4	0.1	4.8	34.3
Quantile .9	56.2	282.3	761.3	0.9	172.4	0.4	16.1	72.6
0	0	0						
length	50							

peak +8	R0	$1/\theta_2$	inf	inflog	newly	newlylog	Reff	AttackRate
mean	26.6	145.8	3292.8	0.9	782.4	0.6	12.1	52.7
median	21.1	107.7	612.	0.8	240.5	0.5	8.5	48.
Quantile .1	8.7	71.2	204.2	0.5	70.8	0.2	3.8	34.5
Quantile .9	53.7	253.	4357.2	1.6	1531.7	1.2	22.2	79.7
4	0	0						
length	50							
